# CELLULAR SENESCENCE AS A THERAPEUTIC TARGET FOR GEROSCIENCE-GUIDED CLINICAL TRIALS

**DOI:** 10.1093/geroni/igz038.2987

**Published:** 2019-11-08

**Authors:** Jamie N Justice, Anoop M Nambiar, Tamar Tchkonia, Nathan K LeBrasseur, Nicolas Musi, Stephen B Kritchevsky, James L Kirkland

**Affiliations:** 1 Wake Forest School of Medicine, Winston-Salem, North Carolina, United States; 2 University of Texas Health Sciences Center at San Antonio and South Texas Veterans Health Care System, San Antonio, Texas, United States; 3 Robert and Arlene Kogod Center on Aging, Mayo Clinic, Rochester, Minnesota, United States; 4 Barshop Institute for Longevity and Aging Studies, Center for Healthy Aging, University of Texas Health Sciences Center at San Antonio and South Texas Veterans Health Care System, San Antonio, Texas, United States

## Abstract

The geroscience hypothesis holds that targeting fundamental mechanisms of aging has the potential to prevent or reduce severity of multiple age-related diseases. Cellular senescence is a key mechanism that may be driving disease in human aging, including Idiopathic pulmonary fibrosis (IPF), a progressive, ultimately fatal, senescence-associated disease. Importantly, cellular senescence may be targeted therapeutically. Senolytic agents are drugs that selectively induce senescent cell apoptosis by transiently disabling anti-apoptotic pathways. Selective ablation of senescent cells using the senolytic drug combination dasatinib plus quercetin (DQ) alleviates IPF-related dysfunction in bleomycin-administered IPF mouse model. We conducted the first-in-human trial of senolytics in IPF patients, and our data indicate that short-term, intermittent administration of DQ may alleviate physical dysfunction that accompanies IPF in human aging, including clinically-meaningfully improvements in mobility (p<0.05). This geroscience-guided clinical feasibility study supports evaluation of senolytics in larger randomized, controlled trials of cellular senescence-associated age-related diseases.

